# Exploring patterns of error in acute care using framework analysis

**DOI:** 10.1186/s12909-015-0285-6

**Published:** 2015-01-16

**Authors:** Victoria R Tallentire, Samantha E Smith, Janet Skinner, Helen S Cameron

**Affiliations:** Centre for Medical Education, University of Edinburgh, Chancellor’s Building, 49 Little France Crescent, Edinburgh, EH16 4SB UK

**Keywords:** Error, Junior doctors, Acute care, Emergencies, Framework analysis

## Abstract

**Background:**

Junior doctors are often the first responders to deteriorating patients in hospital. In the high-stakes and time-pressured context of acute care, the propensity for error is high. This study aimed to identify the main subject areas in which junior doctors’ acute care errors occur, and cross-reference the errors with Reason’s Generic Error Modelling System (GEMS). GEMS categorises errors according to the underlying cognitive processes, and thus provides insight into the causative factors. The overall aim of this study was to identify patterns in junior doctors’ acute care errors in order to enhance understanding and guide the development of educational strategies.

**Methods:**

This observational study utilised simulated acute care scenarios involving junior doctors dealing with a range of emergencies. Scenarios and the subsequent debriefs were video-recorded. Framework analysis was used to categorise the errors according to eight inductively-developed key subject areas. Subsequently, a multi-dimensional analysis was performed which cross-referenced the key subject areas with an earlier categorisation of the same errors using GEMS. The numbers of errors in each category were used to identify patterns of error.

**Results:**

Eight key subject areas were identified; hospital systems, prioritisation, treatment, ethical principles, procedural skills, communication, situation awareness and infection control. There was a predominance of rule-based mistakes in relation to the key subject areas of hospital systems, prioritisation, treatment and ethical principles. In contrast, procedural skills, communication and situation awareness were more closely associated with skill-based slips and lapses. Knowledge-based mistakes were less frequent but occurred in relation to hospital systems and procedural skills.

**Conclusions:**

In order to improve the management of acutely unwell patients by junior doctors, medical educators must understand the causes of common errors. Adequate knowledge alone does not ensure prompt and appropriate management and referral. The teaching of acute care skills may be enhanced by encouraging medical educators to consider the range of potential error types, and their relationships to particular tasks and subjects. Rule-based mistakes may be amenable to simulation-based training, whereas skill-based slips and lapses may be reduced using strategies designed to raise awareness of the interplay between emotion, cognition and behaviour.

## Background

Junior doctors are often the initial responders to patients who become acutely unwell in hospital. It is, however, an area in which junior doctors feel consistently poorly prepared for practice [1]. Previous work has shown that the behaviour of junior doctors in acute care contexts is influenced by a range of interconnected factors [2] and the propensity for error is high [3]. Improved understanding of the errors made by junior doctors within such contexts is therefore pivotal to developing effective educational interventions and improving patient outcomes. The overall aim of this study was to identify patterns of error in acute care, which may subsequently be used to guide the development of targeted educational strategies.

This observational study aimed to build on previous work which examined the validity of Reason’s generic error modelling system (GEMS) in categorising errors made by junior doctors in acute care contexts [4]. As junior doctors rarely work in isolation, the original framework was amplified to include two novel error types which are specific to the team-based nature of acute care provision. The original version of GEMS, along with the additional error categorisations proposed in the aforementioned study are defined and illustrated in Table 1.

**Table 1:** **Definitions, descriptions and examples of the error types described in the amplified version of GEMS** [4,5]

	Error type	Definition	Example from previous work [4]	
			Description of error	Evidence from scenario (S) or debrief (D)
Original GEMS categorisations	Skill-based slips and lapses	“*errors which result from some failure in the execution* [slip] *and/or storage* [lapse] *stage of an action sequence*” [5]	Patient’s notes not checked for current medications as possible cause of hypoglycaemic coma	Junior (D): “*I completely forgot about the kardex* [drug chart], *that’s when I was going to read that he was diabetic, and then the phone went*”
	Rule-based mistakes (RBMs)	“*the mistake arises from the application of a ‘bad’ rule or the misapplication of a ‘good’ rule* [a rule of proven worth]” [5]	Juniors aware that senior help is not arriving for 20 minutes and patient having a major post-operative bleed	Tutor (D): “*Did 2222* [emergency call] *cross your mind?*” Junior: “*Yes it did at one point.*” Tutor: “*Why didn”t you call it?*“ Junior: ”*I felt like the patient’s consciousness wasn’t impaired*.”
	Knowledge-based mistakes (KBMs)	Mistakes arising from “*the more laborious mode of making inferences from knowledge-based mental models of the problem space*” [5]	Recognition of partial airway obstruction but no simple manoeuvres attempted and no advice sought	Junior (S): “*He’s sounding very obstructed; he’s got an obstructed airway*.” Reply from other junior: “*We can’t do anything about it, can we?*”
	Violations	“*Deliberate deviations from those practices deemed necessary to maintain the safe operation of a potentially hazardous system*” [5]	Feels patient’s pulse but does not count rate or ask for any monitoring	Junior (S): “*He’s got a pulse as well; I can’t tell the rate, I don’t have a watch*.”
Novel error types	Compound errors	Errors occurring solely because of a preceding error, from own or others’ misperception or misinterpretation of information	Junior uses observation chart as a surrogate for current physiology and then provides insufficient oxygen to patient	Junior (D): “*We had the patient on a Hudson* [variable performance] *mask… 97% sats* [oxygen saturation] *so I didn’t think we needed to jump in with all guns blazing*.”
	Submission errors	Errors occurring when a junior doctor was dissuaded from taking the most appropriate course of action by a colleague advocating less appropriate measures	Aware patient is bleeding; one junior keen to use blood as primary resuscitation fluid but persuaded by other junior not to request any blood from blood bank	Junior (S): “*I think we should just give more fluid*.” Reply from other junior: “*But if she’s bleeding blood then we should give her blood.*” Junior: “…*can we not just keep giving her saline, or jelly* [colloid] *or something?*”

Whilst the classification of errors occurring in acute care contexts according to the amplified version of GEMS is of academic interest, it is of limited value in developing educational strategies aimed at reducing error. In order to identify educationally-useful patterns within the data, the GEMS classifications need to be cross-referenced with the knowledge, skills and behaviours that are most applicable to the management of acutely unwell patients. The identification of specific patterns of error may facilitate research-informed curriculum design and the development of tailored educational strategies. It seems likely, for example, that the reduction of knowledge-based mistakes necessitates different educational techniques to the reduction of skill-based slips and lapses.

In order for cross-referencing to be undertaken, an appropriate framework or taxonomy encapsulating the subject areas relevant to the assessment and management of acutely unwell patients was required. Several such taxonomies have been developed and utilised in previous studies. Whilst there are clear benefits to the application of a validated framework, the reasons that pre-existing taxonomies were deemed unsuitable are summarised in Table 2.

**Table 2:** **Categorisation, descriptions and limitations of pre-existing taxonomies and frameworks relevant to acute care**

Sub-categorisation of pre-existing frameworks	Description	Limitations in relation to this work
Behavioural marker systems	“*Observable, non-technical behaviours that contribute to superior or substandard performance within a work environment*” [6] which have been sub-divided according to the research-derived categories relevant to a particular context and professional group [7-10].	a) Developed and validated for use within a particular context e.g. The Oxford Non-Technical Skills scale [11] is used in theatre where the challenges clearly differ substantially from those encountered when dealing with a life-threatening situation in a general ward.
		b) Previous studies [12,13] indicate that there are deficits relating to knowledge base and technical skills which need to be identified, in addition to the non-technical skills addressed by behavioural marking systems.
Scenario checklists	Lists of actions or behaviours (often specific clinical tasks) relevant to an individual clinical scenario [14-16].	Most checklists developed for acute care scenarios include aspects of timed assessment (such as time taken to assess airway, breathing and circulation) [16-18] giving numerical values that primarily reveal the consequences and not the causes of error.
Resuscitation competencies	Structured resuscitation courses [19,20] use lists of competencies that have often been developed using a modified Delphi process or similar technique [20].	a) Scenario-specificity combined with granular detail make competency lists unsuitable for this study.
		b) Previous work indicates that whilst technical skills are a source of concern for both junior doctors and their educational supervisors, non-technical skills such as decision-making, initiative and prioritisation are also felt to be important [21].

### Aims

This study aimed to answer the following questions:

1. What are the main subject areas in which junior doctors’ acute care errors occur?
2. How do the errors made in each subject area relate to the types of error as classified by the amplified GEMS framework?

## Methods

### Ethics

Ethical approval for this work was waived by the South East Scotland Research Ethics Committee. Written consent for audio and video data collection and publication of anonymised results was obtained from all participants.

### Design

This study used the data obtained from the simulated acute care scenarios described in a previous study [4]. Due to the practical and ethical implications of observing junior doctors treating acutely unwell patients on the wards, high-fidelity simulation was used to observe junior doctors’ behaviours. Eight simulated scenarios were designed by VRT and two consultant anaesthetist colleagues. After piloting with 16 junior doctors, feedback was sought and the scenarios were refined. The four scenarios considered most reproducible and realistic were used for this study; postoperative haemorrhage, severe sepsis, respiratory distress and hypoglycaemic coma. Each scenario was used with equal frequency.

A full-body adult mannequin simulator (Emergency Care Simulator, Medical Education Technologies, Inc., Sarasota, Florida) was utilised, and was accompanied by the equipment, drugs and paperwork used on the wards where the junior doctors worked. The patient’s voice and physiology (as shown on the bedside monitor and in the mannequin’s respiratory rate) were manipulated from the control room. A telephone present in the simulation room connected directly to the control room. A member of staff unknown to the participants played the role of a ward nurse and provided accurate information when requested but did not actively prevent errors.

Thirty-eight junior doctors (representing recent graduates of seven different UK medical schools) were recruited on a volunteer basis. They were briefed regarding room layout, nurse capabilities and mannequin features and limitations. They then participated in a total of 18 simulated scenarios in groups of two or three and were asked to treat the patient (mannequin) as they would do on the ward. A facilitated debrief focusing on the cognitive aspects of decision-making occurred immediately after each scenario. The debriefs were conducted by one of three trained senior clinicians (VRT and two consultant anaesthetists). Each debrief involved playback of video from the scenario and encouraged articulation of the cognitive processes. Debriefs were audio recorded and field notes were taken by either VRT or SES.

Evidence from the video-recorded scenarios, audio-recorded debriefs and field notes were used to list all of the errors made in each scenario. These errors were classified according to the amplified version of GEMS, as described previously [4].

### Inductive development of key subject areas

The first research question was addressed by using the principles of ‘framework analysis’ to inductively develop a thematic framework consisting of key subject areas [22]. Originally developed within the field of applied social policy research, ‘framework analysis’ is an analytical process which facilitates systematic analysis of qualitative data whilst promoting the generation of “actionable outcomes” [22]. During the preliminary stage of this work, VRT and SES noted brief descriptions of each of the errors that were identified. Using a combination of these descriptions and the intention-related evidence derived from either the video-recorded scenario or audio-recorded debrief, VRT and SES inductively developed a preliminary thematic framework. As expected, the first version of the framework drew heavily on previous related work [2], and other “a priori issues” [22]. VRT and SES then independently applied the early version of the framework to the list of errors obtained from the first four scenarios, allowing the developing framework to be influenced by emergent issues and analytical themes arising from the recurrence of particular error types. VRT and SES then discussed their independent analyses and compared, contrasted and negotiated categories of errors until agreement on a final indexing system was reached.

### Application of thematic framework

Once finalised, the thematic framework was systematically applied to the entire dataset of junior doctor errors (i.e. the full list of errors obtained from the initial analysis of all 18 scenarios). Working together, VRT and SES discussed the error descriptions from the video-recorded scenarios in conjunction with the additional evidence derived from the scenarios (such as direct quotes, body language and other non-verbal clues) and debriefs (including direct quotes and other paralinguistic clues such as laughter), until agreement on categorisation was reached. The use of Excel (Microsoft Office 2007) for the indexing of errors facilitated inter-scenario and intra-scenario comparison of errors so that patterns within the dataset as a whole could be identified and explored.

### Pattern identification

In order to address the second research question, a multidimensional analysis involving both the amplified GEMS classifications [4] and the inductively-developed subject areas was undertaken. In keeping with the principles of framework analysis, a distilled summary of each error was entered into a chart to promote abstraction and synthesis [22]. Throughout the analysis, each error remained referenced with a specific numerical code so that the source scenario could be traced and contextual validity continually checked. The errors within an individual subject area were then compared and contrasted, and patterns within the data were sought.

Patterns were identified by counting the number of errors that occurred in relation to each subject area and GEMS classification. The use of numbers in qualitative research is a controversial issue. Most qualitative researchers who reject the use of numerical data articulate their objections with reference to the philosophical underpinning of their work. Maxwell (2010) states, “Primarily, this is because they have believed that numerical data are incompatible with a constructivist stance for research, as such data imply the existence of a single “objective” reality that can be measured and statistically analysed to reach generalisable conclusions” [23]. However, several prominent qualitative researchers have supported the inclusion of numbers in qualitative research practices and reports for many years [24,25], and the discipline of medical education is beginning to embrace the concept [26]. This study was undertaken on the premise that the use of numbers alone does not define the difference between constructivist and positivist research paradigms. The incorporation of numerical data in this work helps to reveal patterns, provide precision and promote clarity. They have, however, been used only in ways that recognise their limitations, preserve the richness of the dataset and do justice to the complexity of the phenomena being studied.

## Results

Eight key subject areas formed the final version of the thematic framework: hospital systems, infection control, prioritisation, procedural skills, situation awareness, treatment, communication and ethical principles in practice. The number of errors relating to each subject area, sub-classified using the amplified version of GEMS, is displayed in Table 3. The purpose of Table 3 is to allow comparison of the different error types *within*, as opposed to *between*, the various subject areas. It is the patterns within the data, as opposed to the actual numerical values, that are of interest. Table 4 illustrates specific examples of errors relating to each of the key subject areas and details the associated GEMS classifications.

**Table 3:** **A multidimensional analysis of errors categorised according to both the amplified version of GEMS and the inductively-developed key subject areas**

Key subject area	Amplified version of Reason’s generic error modelling system (GEMS)						Intention not established	TOTALS
	Skill-based slips/lapses	Rule-based mistakes	Knowledge-based mistakes	Violations	Compound errors	Submission errors		
Hospital systems	1	**24**	13	3	0	0	11	52
Infection control	1	0	0	0	0	0	**18**	19
Prioritisation	1	**10**	0	3	0	2	6	22
Procedural skills	**18**	0	**12**	0	1	0	3	34
Situation awareness	**20**	9	8	1	**19**	1	11	69
Treatment	2	**12**	6	0	0	1	2	23
Communication	**11**	0	1	1	1	1	2	17
Ethical principles in practice	0	**6**	0	1	0	0	0	7
TOTALS	54	61	40	9	21	5	53	243

The bold numbers indicate patterns within the dataset.

**Table 4:** **Specific examples of errors relating to seven of the key subject areas**

	Description of error ( *scenario number in parentheses* )	Evidence from scenario (S) or debrief (D)	GEMS classification
**Hospital systems**			
1	Surgeon paged (but had not answered) and junior doctors assumed that the surgeon was therefore on his/her way to the ward (2)	Junior (S): “*He’s been called so he’s on his way*.”	Rule-based mistake
2	Patient with major post-operative bleeding is causing concern but no attempt made to obtain senior help (17)	Junior (D): “*I was thinking about maybe calling the anaesthetist. I was thinking: I need an anaesthetist, where do I get one of those?’*”	Knowledge-based mistake
**Prioritisation**			
3	Specific investigation (electrocardiogram [ECG]) is arranged before any assessment of the patient has been undertaken (3)	Junior (S): “*What we need to do first is another trace of the heart*.”	Rule-based mistake
4	One junior doctor is very keen to call for senior help but dissuaded from doing so by other junior who insists on the requirement for investigation results prior to calling (9)	Junior (S): “*Should we get an SHO* [more senior doctor] *here?*” Reply from other junior: “*I suppose we need to send the bloods first, and get an ECG* [electrocardiogram].”	Submission error
**Procedural skills**			
5	Nurse corrects lead placement of junior doctor for ECG monitor (6)	Nurse (S): “*The red one goes on the other side*.” Junior: “*Oops, so it does*.”	Skill-based slip/ lapse
6	Recognition of severe sepsis but no attempts made to give antibiotics (18)	Tutor (D):“*Did the patient get antibiotics?*” Junior: “*No, because I didn’t know how to administer them*”	Knowledge-based mistake
**Situation awareness**			
7	Junior doctor suggested checking the volume of blood in the patient’s drains, but the task was never undertaken (12)	Junior (D): “*I remember you saying ‘have you checked the drains?’ because we hadn’t.*” Other junior doctor: “*but then I didn’t actually myself look at the drains when I should have, I thought you had, yeah, I thought…*”	Skill-based slip / lapse
8	Junior doctor tells senior colleague on the phone that a 12 lead ECG has been performed when it has not, it had merely been mentioned to the nurse	Junior (D): “*When she was asking me what tests we had done and for information on what we’d done, you know, we seemed to have covered all the bases.*”	Compound error
**Treatment**			
9	Patient in septic shock with no evidence of cardiac dysfunction treated with 500mls of saline over one hour (3)	Junior (S): “*I don’t want to put him into heart failure, let’s put it over an hour.*” [discussing intravenous fluid prescription with nurse]	Rule-based mistake
**Communication**			
10	During phone call, surgical registrar [more senior doctor] is dismissive of junior doctor, who is told to ‘just carry on’ but left with the false impression that the senior doctor was coming to help (5)	Junior (D): “*I felt better because they* [the surgical registrar] *were coming to see the patient.....if I had been completely useless in my handover then they probably would have just said for me to do all these tests and then ring back…*”	Skill-based slip/ lapse
**Ethical principles in practice**			
11	Junior doctors persuaded by hypoxic, confused, exsanguinating patient to remove the oxygen mask (9)	Junior (D): “*I didn’t know how much you can make someone do something who is, you know, confused. But then he’s sick. That was hard*.”	Rule-based mistake

### Summary of error patterns

In relation to **hospital systems**, there was a predominance of rule-based mistakes, with many errors related to attempts to obtain senior assistance. These errors often involved a misunderstanding of the purpose of certain procedures or protocols, and frequently involve application of a ‘bad’ rule. As shown in Table 3, the same pattern was observed in relation to errors of **prioritisation**. Rule-based mistakes commonly involved junior doctors deciding to undertake investigations, such as an electrocardiogram, prior to assessing the patient’s airway patency. In relation to **procedural skills**, most errors were skill-based slips or lapses, commonly involving failure to remove the tourniquet from the patient’s arm following intravenous cannula insertion. The predominance of slips and lapses is likely to be attributable, at least in part, to the psychomotor aspects of procedural skills. Many of the errors that could be attributed to a lack of **situation awareness** were skill-based slips or lapses stemming from interruptions during the initial clinical examination. There were also a large number of compound errors originating from the misunderstandings of others, as well as from a junior doctor’s own misperception of information.

**Treatment** errors were commonly rule-based mistakes related to type or flow rate of intravenous fluid resuscitation or antibiotic choice. In contrast, **communication**-related errors were mainly skill-based slips and lapses that involved mishearing or misinterpreting verbal information provided by the nurse helper, or misinterpreting what was said in a telephone conversation. In relation to **ethical principles in practice**, rule-based mistakes most commonly occurred when the capacity of the patient to refuse life-saving treatment was impaired due to critical illness, but potentially life-saving treatment was withheld or even withdrawn as a result of overarching concern for patient autonomy. There were insufficient data to elucidate the causes of error in relation to **infection control**.

## Discussion

This study has built on previous work by using the amplified version of GEMS, in combination with iteratively-derived key subject areas, to explore and classify the types of errors made by junior doctors in acute care contexts. The results provide a springboard for the deeper consideration of specific errors types, their origins within medical training and potential educational strategies aimed at reduction of error and improvement of patient outcomes.

The finding that prioritisation was a key subject area in which rule-based mistakes were commonly made echoes previous work concluding that prioritisation is a key component of a junior doctor’s role which is usually learned ‘on the job’, making doctors in their early days feel unprepared [27,28]. A focus group study of junior doctors’ behaviour in acute care contexts has previously described the difficulties that newly qualified doctors face when attempting to transfer knowledge into practice [2], particularly in relation to applying a structured approach to patient assessment [2]. In acute care, popular assessment structures (such as ABCDE: airway, breathing, circulation, disability, exposure) and standardised protocols can make prioritisation of tasks easier. However, a high level of familiarity with such structures is required to recall and utilise them in times of acute stress. Primary medical training programmes could tackle this issue by facilitating the repeated rehearsal of basic patient assessments in a variety of contexts, to emphasise the transferability of such assessment structures. This learning is amenable to simulation training, whereby students can experiment with changing priorities whilst observing and subsequently discussing the clinical consequences. However, care must be taken in the planning and execution of such training to replicate the complexities and pressures of the environment in which clinical decisions will ultimately be made. The decontextualised rehearsal of basic assessment structures in simulation training may actually hinder educational development and, if trained in this way, junior doctors are likely to continue to have difficulty utilising such knowledge in the stressful and hierarchical world of clinical practice [29,30].

In contrast to prioritisation errors, procedural skills were strikingly vulnerable to slips and lapses. It is likely that the prevalence of slips and lapses in relation to procedural skills is, at least in part, influenced by the stressful nature of acute care [2]. Elevated stress levels have been shown to impede performance in a multitude of cognitive processes required in acute care contexts including those that involve divided attention, working memory, retrieval of information from memory, and decision making [3]. Furthermore, the results of this study demonstrate that the undertaking of a procedural skill within an acute care scenario predisposes to the common tendency for attention to become so focussed on one aspect of a situation that other important cues go unnoticed [3,31]. It is therefore important that junior doctors are aware of the interplay between emotion, cognition and behaviour, and the roles of such factors in errors and adverse events. Emotional skills training, particularly with reference to dynamic, high-stakes situations, might help to facilitate this learning. Such training should acknowledge the influence of stress and provide strategies to reduce its impact. Another approach to tackling the problem of slips and lapses whilst performing procedures would be to utilise educational techniques that involve distraction. Techniques can be developed which specifically aim to enhance performance of basic procedures safely and effectively whilst deploying attention elsewhere. Based on automaticity theory [32], the gradual additions of distraction or time–pressure to the rehearsal of practical procedures are useful strategies that are beginning to be explored within the field of healthcare education [33]. It therefore seems likely that carefully designed simulation-based training has the potential to expose and address multiple error types, including those related to both prioritisation and procedural skills.

### Strengths and limitations

This study used high-fidelity simulation to explore patterns of error in acute care. Observation through video is an under-utilised research method [34] that has the advantage of capturing linguistic, paralinguistic and non-verbal communication. The inductive development of a novel framework has the advantage of maintaining the richness of the dataset, and the use of framework analysis has facilitated the generation of actionable outcomes. However, the study has several important limitations. Particular difficulties were associated with identifying all of the errors contained within the scenarios, and the identification process was undoubtedly influenced by the ideas, beliefs and clinical experience of the researchers. Some of the errors categorised into one key subject area could arguably be classified into another if slightly different definitions had been adopted. It is also likely that, given the complexity and somewhat subjective nature of the analysis, alternative researchers would have coded some errors differently. Furthermore, the lack of sufficient evidence to attribute 53 of the errors to a single cause necessitated their exclusion from the multidimensional analysis, as detailed in Table 3.

A major limitation of all studies employing simulation is that behaviour in simulated environments may not mimic behaviour in everyday clinical practice. In the context of this work, this seems particularly likely in relation to certain key subject areas, such as infection control, where the absence of a real sense of infection risk may have influenced the decision to wear gloves for infection-prone procedures. These limitations were minimized by the use of high-fidelity simulation involving fake blood and genuine wound dressings, but could not be entirely eliminated. Infection control errors were rarely explored during debriefing and consequently there was usually insufficient evidence to confidently attribute each infection control error to one of a number of possible explanations. It is interesting to consider whether tutor suspicion of ‘simulator artefact’ was the explanation for this lack of emphasis during debriefing. The error pattern within this key subject area has therefore not been established using this method. In addition, the presence of a nurse helper who always provided information that was accurate and relevant may not reflect the clinical workplace. It is likely that, despite their best intentions, nurses and other professionals may, at times, actually contribute to error generation, particularly compound and submission errors.

### Future work

The patterns of error identified in this study could be used to explore some specific educational strategies (as discussed above) designed to reduce error in acute care. The impact of such strategies on the subsequent behaviour of junior doctors needs to be carefully examined, perhaps using simulated environments. Furthermore, analyses such as the one detailed here could be used to provide information on the shortfalls of individual primary medical degree programs, and the impact of curricular changes. Similar methods could also be used to delineate the types of error most prevalent in other contexts or professional groups, in the hope that tailored education innovations will be more effective at reducing error than generic teaching.

## Conclusions

For the initial assessment and management of acutely unwell patients by junior doctors to be improved, it is important that medical educators understand the causes and patterns of common errors. Adequate knowledge alone does not ensure prompt and appropriate management and referral. Acute care skills education may be enhanced by encouraging medical educators to consider the range of potential error types, and their relationships to particular tasks and subjects. In conjunction with process review and system redesign, it is hoped that novel teaching strategies may be developed and implemented, enhancing the performance of junior doctors and the safety of acutely unwell patients.

## Abbreviations

GEMS: Generic error-modelling system
RBM: Rule-based mistake
KBM: Knowledge-based mistake
